# A hybrid mask RCNN-based tool to localize dental cavities from real-time mixed photographic images

**DOI:** 10.7717/peerj-cs.888

**Published:** 2022-02-18

**Authors:** Umer Rashid, Aiman Javid, Abdur Rehman Khan, Leo Liu, Adeel Ahmed, Osman Khalid, Khalid Saleem, Shaista Meraj, Uzair Iqbal, Raheel Nawaz

**Affiliations:** 1 Department of Computer Science, Quaid-e-Azam University, Islamabad, Pakistan; 2 School of Business and Law, The Manchester Metropolitan University, Manchester, United Kingdom; 3 Department of Computer Science, COMSATS University, Islamabad, Pakistan; 4 Department of Radiology, Bolton NHS Foundation Trust, Bolton, United Kingdom; 5 Department of Computer Science, National University of Computer and Emerging Sciences, Islamabad Chiniot-Faisalabad, Pakistan

**Keywords:** Dental cavities, Deep learning, Dental image processing, Mask RCNN

## Abstract

Nearly 3.5 billion humans have oral health issues, including dental caries, which requires dentist-patient exposure in oral examinations. The automated approaches identify and locate carious regions from dental images by localizing and processing either colored photographs or X-ray images taken *via* specialized dental photography cameras. The dentists’ interpretation of carious regions is difficult since the detected regions are masked using solid coloring and limited to a particular dental image type. The software-based automated tools to localize caries from dental images taken *via* ordinary cameras requires further investigation. This research provided a mixed dataset of dental photographic (colored or X-ray) images, instantiated a deep learning approach to enhance the existing dental image carious regions’ localization procedure, and implemented a full-fledged tool to present carious regions *via* simple dental images automatically. The instantiation mainly exploits the mixed dataset of dental images (colored photographs or X-rays) collected from multiple sources and pre-trained hybrid Mask RCNN to localize dental carious regions. The evaluations performed by the dentists showed that the correctness of annotated datasets is up to 96%, and the accuracy of the proposed system is between 78% and 92%. Moreover, the system achieved the overall satisfaction level of dentists above 80%.

## Introduction

The orally transmitted infectious diseases, especially the COVID-19, have presented the world with unanticipated severe challenges (Singer, 2020). The healthcare workers are most affected since they have direct exposure to the patients. Many essential healthcare practices are closed to impose social distancing in the population (Vaccaro et al., 2020). Dental practitioners are also affected due to having a higher risk of infection transmission (Bhumireddy, Mallineni & Nuvvula, 2020). Oral dental health is crucial and essential for overall well-being (Kane, 2017; Peres et al., 2019). At present, researchers are trying to detect tooth caries from dental images by localizing carious and non-carious regions (Navarro et al., 2019).

In the literature, a dental cavity can be described as the destruction of a tooth’s tissue (Manjunatha, 2013). Dental caries are becoming a widespread problem causing global suffering (Kane, 2017; Peres et al., 2019). According to the World Health Organization (WHO) report (https://www.who.int/news-room/fact-sheets/detail/oral-health), 100% of young adults and 60–90% of children have at least one carious region. Adults aged between 35–44 face severe gum diseases. Moreover, 30% of the overall world population above 65 years have lost their natural teeth (Petersen et al., 2010). The dental caries identification by dentists at early stages may protect the patients from severe dental damages (Gugnani et al., 2011).

Dental issues require proper consultancy from dentists who follow the traditional protocols in disease diagnosis (Navarro et al., 2019; Petti, Glendor & Andersson, 2018). Usually, dentists resort to standard procedures, such as mirror explorer, colored images photographs, and X-ray radiographs, to manually inspect dental issues. However, manual diagnostic methods may sometimes lead to critical errors or misdiagnosis. Artificial intelligence and deep learning advancements motivated researchers to develop useful tools to detect carious regions from dental images. The colored dental photographs (captured *via* digital cameras) are used to capture the early lesions, whereas X-ray radiographs are usually used to capture severe dental cavities (Volinski, 2016).

The existing dental carious regions’ localization approaches usually employ colored photographic or X-ray radiographic datasets (Navarro et al., 2019). However, the recent work mainly focuses on neural network models to localize the carious region (Navarro et al., 2019). It masks the detected carious region with a solid block of color that omits the dental caries details, making it hard for physicians to interpret (Gonsalves, 2017). A tool to detect dental caries from colored photographs, X-ray radiographs, and mixed (*i.e*., colored photographs and X-ray radiographs) is yet to be discovered.

Researchers have proposed an approach to locate the carious region in X-rays radiographs (Gonsalves, 2017). However, due to the lack of experimental details, the performance of this approach is not verified. Therefore, a hybrid approach must be introduced to locate the carious region in bounding boxes in dental colored photographs (taken *via* a camera), X-ray radiographs, and mixed (colored photographs and X-ray radiographs) images. The hybrid approach may locate the dental carious regions from both types of dental images. It may ease the dentist to detect dental caries and help in avoiding the dentist’s exposure to the patients in detecting the early lesion. Our previous research introduced a deep learning approach to localize carious regions from photographic dental images taken *via* high-definition cameras (Javid, Rashid & Khattak, 2020). The existing research is mainly based on colored dental photographs focusing on the elementary pre-processing steps, significant validations, and the effectiveness of M-RCNN. The study yields satisfactory results. The accuracy of the M-RCNN reaches more than 90% (Moutselos et al., 2019).

This research proposed a localization approach to locate the carious region from colored photographs, X-ray radiographs, and mixed dental images (*i.e*., colored photographs and X-ray radiographs) by exploiting the M-RCNN model. Our approach further draws rectangular boxes around the carious regions to ease locating the carious area. The proposed approach is efficient and can run on a minimum hardware configuration. We employed locally collected and annotated dental images from Combined Military Hospital (CMH) Rawalakot, Pakistan, along with standard datasets in training and validation. The annotation involves the selection of dental images (irrespective of age and cavity type), image quality enhancement, and image carious regions labeling by the experts.

In addition to this, we also instantiated an M-RCNN based dental cavities localization tool to facilitate practitioners in diagnosing carious regions with less exposure to patients. The tool also enables novice users to self-diagnose caries at home by uploading the dental photographs (taken *via* their ordinary cameras at home) on the tool. The dentist can get dental photographs in a controlled clinical setting or from the patients that can be further employed in dental cavities preliminary investigation. Hopefully, this may involve less direct dentist-patient exposure. In a way, the tool lessens coronavirus contact caused due to detailed dental examination in typical clinical settings. The following is the summarized list of contributions.

- We collected and annotated multiple types of dental images in the dataset, *i.e*., colored photographs, X-ray radiographs, and mixed dental images.
- We defined and modeled a hybrid deep neural network-based M-RCNN to localize the dental carious regions for colored photographic and X-ray radiographic images.
- A comprehensive architectural design is presented to instantiate a software tool that can localize the carious regions from multiple types of dental images.
- We implemented a software-based dental caries detection tool to localize the dental carious regions to support the dentist and the patients at preliminary diagnosis stages.

The rest of the paper is organized as follows. In “Literature Review”, we discuss the literature review and motivation. “Materials and Methods” elaborates the materials and methods used in the research. In “Experimental Setup and Results”, we explain the experimental setup and results. “Comparison and Implications” provides the comparisons with state-of-the-art and implications of the proposed research. Finally, in “Conclusion and Future Work”, we conclude our discussion and highlight the future research directions.

### Literature review

The recent advancements in artificial intelligence and deep learning techniques reduced human efforts and error rates in dental caries localization (Volinski, 2016). The clinical practices employed various machine learning approaches in dental caries diagnosis (Prados-Privado et al., 2020). The dental carious regions’ localization approaches use image processing techniques, classifiers, neural networks, *etc*., to detect faulty dental regions (Jader et al., 2018; Ezhov et al., 2021). In addition to this, researchers have proposed dental caries localization tools. We will discuss dental caries localization approaches and the tools in the following. We also discuss state-of-the-art dental caries detection and treatment techniques, particularly in a clinical context.

### Dental caries localization approaches

The dental caries localization approaches use a variety of image types to diagnose carious regions in clinical settings. The image type includes but is not limited to panoramic images, ultrasound images, bitewing radiographs, colored photographs, X-ray images, *etc*. (Kumar, Bhadauria & Singh, 2021). The employment of panoramic X-ray images in the localization of dental images is imprudent of imaginary surrounding areas (chin, spine bones, jaws, *etc*.) (Lee et al., 2018). The localization requires multiple images in a single dental cavity localization (Liang et al., 2020). The existing research also shows less satisfactory results in dental cavities detection *via* X-ray panoramic dental images due to their complexity (Dibeh, Hilal & Charara, 2018). The panoramic radiographs may cause mistreatment due to misdiagnosis in some instances (Endres et al., 2020). Utilizing ultrasound images to local dental cavities *via* deep learning approaches requires vast expertise in scanning and ultra-sound operations (Nguyen et al., 2021). Bitewing radiographs show less sensitivity for a variety of services, including proximal and occlusal. Their performance may not be satisfactory in detecting non-cavitated lesions (Chen, Stanley & Att, 2020). Exploiting machine-readable intra-oral dental photographs (colored) taken *via* high-resolution cameras gives accurate results (Kühnisch et al., 2021). Recent literature is evidence of the employment of colored photographs taken *via* smartphones in dental cavities detection. The localization sensitivity is between 75% and 100%, particularly in sound teeth and extensive lesions (Bayraktar & Ayan, 2021). Recent research also shows that the X-ray radiographs provide dental carious region detection accuracy even in the case of small datasets (Rad, Rahim & Kolivand, 2018).

The employment of colored and X-ray images is less challenging than other image formats since the mentioned image types are less complex and widely available in clinical settings. The following sub-sections discuss the existing schemes and underlying machine learning models, which are mainly employed to detect carious regions from colored photographs and X-ray radiographs.

### Localization *via* colored photographs

Koutsouri et al. (2013) proposed a non-supervised method to detect carious regions. The authors applied a k-means clustering algorithm to segment caries and extracted features from RGB channels. Human experts annotated the images. Vashishth, Kaushal & Srivastava (2014) proposed an approach to detect dental caries in radiographic and intra-oral images. The authors converted the colored images into black and white and used a Gaussian filter to enhance the image quality. The authors segmented each image into various segments and further classified carious images by exploiting threshold computed *via* black pixels. The mentioned approach mainly spotted the black regions as dental cavities. Berdouses et al. (2015) developed a model to detect the pit and fissure cavity from colored images. The authors worked on premolars and molars and eventually converted the 100 colored images to grey-scale images. The authors used the k-means algorithm to eliminate the uninterested areas. Moreover, the authors further employed j48, Random Tree, Random Forests, SVM, and Naive Bayes classifiers to detect carious images. Ghaedi et al. (2014) proposed an automated cavity detection and scoring system. The authors worked on optical colored images and used histogram-based contrast enhancement. The entropy, gradient, and matrix elements were extracted and used in random forest to classify the images. Navarro et al. (2019) proposed a model to localize the smooth surface cavity by considering only colored dental images. The histogram equalization was done pre-processing, and the image was segmented further into 10 × 10 blocks. The intensity, gradient, hue, saturation, and entropy features were extracted from each block. The authors used SVM and decision trees as classifiers.

### Localization *via* X-ray images

Oprea et al. (2008) classified only enamel, dentin, and pulp caries using rule-based classification to detect the tooth edge. The carious region was identified by calculating the width of the cavity and adjacent black-white pixels. The authors categorized regions of size more than 2 mm as dentin caries. Datta & Chaki (2015) proposed a model to detect the cavities using a Wiener filter. The authors segmented the images using a cluster-based segmentation technique. The authors extracted the regions as caries containing many black pixels lacking the detection of carious lesion shape and depth. ALbahbah, El-Bakry & Abd-Elgahany (2016) proposed an approach to detect dental caries using a dataset containing 100 panoramic images. The carious regions were segmented based on a threshold, and a gradient histogram was extracted from the images. Finally, an SVM classifier was used to classify the carious and non-carious teeth. Prajapati, Nagaraj & Mitra (2017) proposed a model to classify dental diseases using Convolutional Neural Network (CNN) and transfer learning. The authors used the VGG16 pre-trained model to classify dental diseases, such as dental caries, periapical infection, and periodontitis. Naam et al. (2017) proposed a methodology to identify dental caries using 27 X-ray panoramic images covering the whole mouth area. The tooth regions were manually extracted by cropping the images. They extracted gradient and intensity features from the images and performed SVM classification to classify the carious and non-carious dental images. Jader et al. (2018) proposed a technique to segment 1,224 X-ray images. They used R-CNN to segment the tooth regions, used the classifier named ResNet101.

Most of the existing X-ray based approaches exploited the low-level image features in dental caries localization. However, a research gap exists in deep learning approaches to localize the dental cavities in bounding boxes and solely classify the carious regions. The mentioned approaches usually employ colored photographic or X-ray radiographic images to local dental carious regions. We discuss dental caries localization *via* state-of-the-art deep learning approaches in the following.

### Dental caries localization: deep learning approaches

Lee et al. (2018) investigated the Deep CNN models to detect and diagnose caries *via* periapical radiographs of molars, premolars, and molars. Cantu et al. (2020) detected caries lesions *via* a convolutional neural network (U-Net) by employing bitewing dental radiographs. They found *via* experimentation that deep learning may facilitate the dentist to detect and locate caries more efficiently (Cantu et al., 2020). Zhang et al. (2020) employed a deep learning-based system containing a CNN to detect dental caries from oral photographs. They found convolutional neural network promising over dental photographs captured *via* ordinary consumer cameras (Zhang et al., 2020). Casalegno et al. (2019) trained the CNN model to automate the detection and localization of early-stage dental lesions/caries from radiographic transillumination (TI) images. Karimian et al. (2018) presented the CNN model trained over the optical coherence tomography (OCT) imaging modality to classify the oral tissues of humans and detect the early dental caries. They also validated the approach over OCT images of enamels, cortical bones, trabecular bones, muscular tissues, and fatty tissues (Karimian et al., 2018).

### Dental caries localization: tools

A few software exists for caries detection and localization. For instance, Logicon caries detection software is used to detect proximal caries (Bentsen, Svensson & Wenzel, 2001). Proximal caries exists in the distal and mesial surface of the tooth. The software enhances the dental X-ray radiographs and highlights the carious region. However, the tool is less efficient than human observers (Bentsen, Svensson & Wenzel, 2001; Wenzel, 2002). Curve Dental (https://www.curvedental.com/) is an automated mid-level tool preferable for dental practitioners. The tool is comprehensive and considers everything from imaging to patient education to scheduling and billing. The SOTA (https://www.curvedental.com/). Image Software is specially designed to handle dental imaging. Practitioners use high-quality colored photographic images of all sizes for training purposes. It mainly adjusts image filters to localize carious regions. The software reduces the amount of time needed to assist the dental education training. Tab32 (https://www.tab32.com/) is cloud-based dental software consisting of a modern interface in growing dental offices. Many practitioners can analyze dental imaging, maintain electronic health records and patient communication. Generally, the localization tools enable the manual labeling of dental carious regions. DIAGNOcam (https://www.kavo.com/dental-xray-machines-and-diagnostics/diagnocam-diagnostic-devices) is an integrated camera-oriented tool that detects the early enamel and dentin caries directly from the captured dental photographs (Casalegno et al., 2019).

### Motivation

The existing approaches, traditional or advanced, handle a single dental image type, *i.e*., colored photographs, or X-ray radiographs. To the best of our knowledge, the detection and localization of dental carious regions in rectangle boxes to enable further exploration by dentists *via* multiple image types is not yet common. Mainly the techniques provide the binary classification of dental images (Sornam & Prabhakaran, 2017). It becomes difficult for dentists to find carious regions in the dental images due to the lack of usable carious region detection and localization tools. The deep learning approaches that extract high-level image features and localize the dental image carious regions are discussed in the literature, primarily focusing X-ray based radiographic images (Milošević et al., 2022; Morid, Borjali & Fiol, 2021). Moreover, a functional tool to detect and localize dental carious regions is unavailable for mixed images, *i.e*., colored photographs and X-ray radiographs. This research proposed a hybrid approach that works with both colored photographic and X-ray radiographic dental images using the M-RCNN model.

## Materials and Methods

### Dataset collection and annotation

To the best of our knowledge, there is no publicly available mixed annotated dataset containing colored photographs and X-ray radiographs of dental caries. Similarly, the raw datasets containing dental carious regions are also inadequately available and fewer in quantity. The existing datasets limit the research in dental cavity localization approaches. To facilitate future cavity localization approaches, we tried to provide a publicly available mixed dataset containing annotated colored photographic and X-ray radiographic images. We formed an archive of 936 manually annotated X-ray radiographs’ datasets collected in a significant proportion from a hospital. A written consent was obtained from all the participants. In addition, a collection of 90 manually annotated colored photographic dataset is created. The dataset is available at Zenodo repository (https://doi.org/10.5281/zenodo.5680365). The dataset can be used publically since permission is granted from the CMH Rawalakot (included in the Zenodo repository). The dataset details are given in Table 1.

**Table 1:** A summary of the dataset containing colored photographic, X-ray, and mixed dental images.

	Image type	No. of instances	Source
${\rm D}{{\rm S}_1}$	Colored	90	Digital lab Spain Granada.
${\rm D}{{\rm S}_2}$	X-ray	936	120 images from Vahab Archives and 816 images from CMH AJK Rawalakot.
${\rm D}{{\rm S}_3}$	Mixed	210	Mixing of DS1 and 120 images from Vahab Archives.

The dataset $D{S_1}$ contains annotated colored photographic dental images, accessible from the Digital Imaging Library of the Optics Department Granada University, Spain. This dataset consists of color dental photographs taken with a Canon EOS 7D digital single-lens reflex color camera with a 135 mm standard lens. Moreover, the dataset constitutes different resolution and file format photographs. The aforementioned database is publicly available under a standard license and used by many researchers (Datta & Chaki, 2015; ALbahbah, El-Bakry & Abd-Elgahany, 2016). We employed *jpg* file format because the *tiff* file format is larger and increases processing time (Rosado et al., 2020). Moreover, we used Set-7 and Set-8, containing photographs of dried oral caries (Eckhard et al., 2012). Collectively, this dataset consisted of 90 colored photographic images. The dataset $D{S_2}$ contains annotated dental X-ray radiographs collected from various sources. We used publicly available Vahab Archive (https://mynotebook.labarchives.com/share/Vahab/) containing 120 X-ray images (Koutsouri et al., 2013). We also locally collected 816 X-ray images from CMH, Rawalakot, Pakistan. The locally managed X-ray images dataset was merged with the Vahab archive dataset. It collectively increased our X-ray dataset to a total of 936 dental X-ray images.

The dataset $D{S_3}$ also contains mixed annotated dental images. It consists of publicly available colored photographic and X-ray radiographic images, including 90 photographic colored images collected from Digital Imaging Library Granada Spain and 120 dental X-ray images from Vahab Archive. We did not include the locally collected X-ray radiographs to balance the ratio of colored photographs and X-ray radiographs for accurate training and testing of the proposed M-RCNN model. It is noteworthy that a significantly unbalanced ratio of training images can cause the model overfitting issues and may lead to biased results (Ren et al., 2012). The mixed dataset collectively consists of 210 images belonging to different types.

### Localization approach

#### Overview

**Figure 1:**
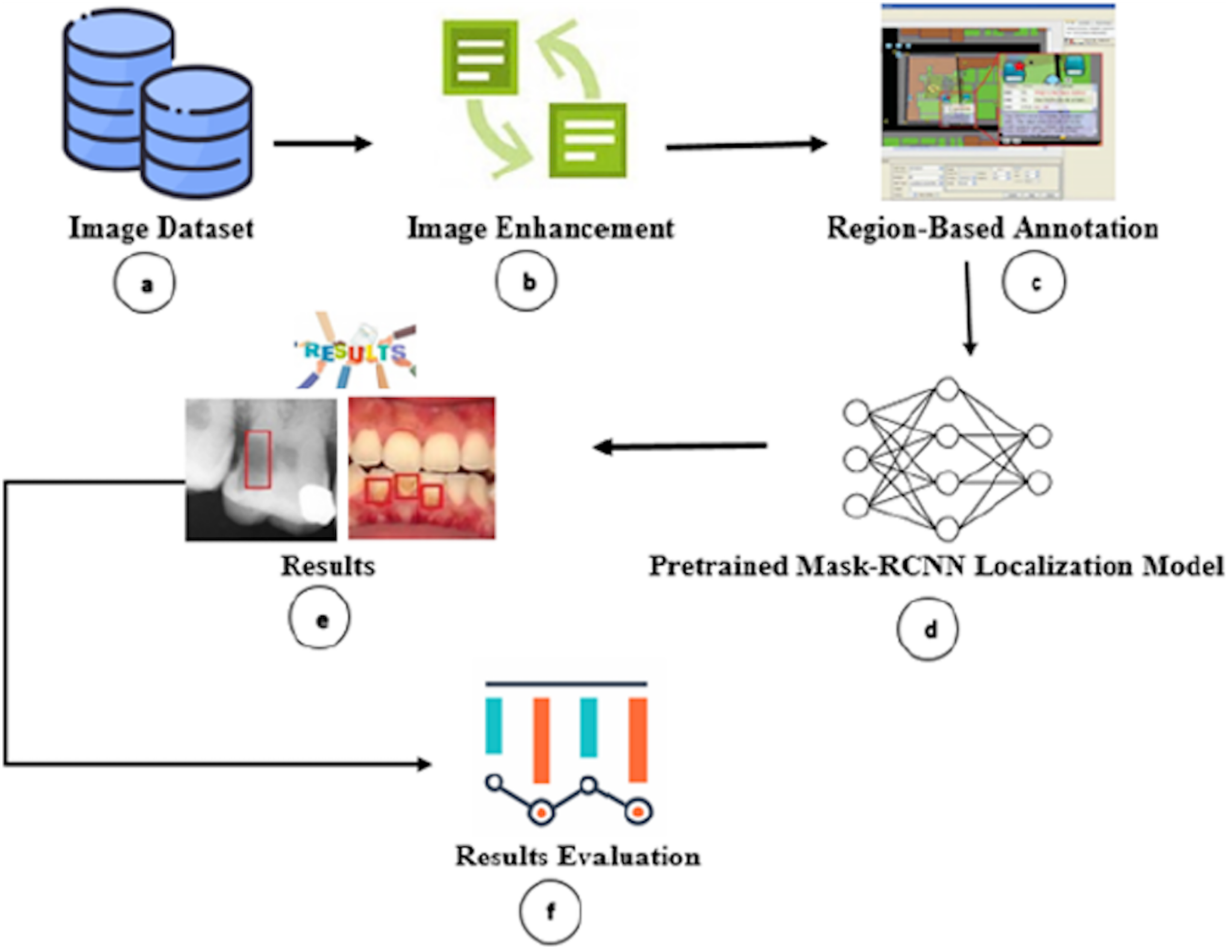
Overview of proposed carious regions detection and localization.

We proposed a transfer learning approach consisting of six major phases, as shown in Fig. 1.

Firstly, we gathered and annotated the dental colored photographs, X-ray radiographs, and mixed (*i.e*., colored photographs and X-ray radiographs) dataset (Fig. 1A). Secondly, we performed image enhancement to improve the image quality for improved carious region detection (Fig. 1B). Thirdly, we annotated the carious regions *via* human dental experts to train our model (Fig. 1C). Fourthly, we passed the annotated images to M-RCNN model (Fig. 1D). The M-RCNN learns the carious tooth regions from the annotated images for autonomous detection. The M-RCNN neural network enables proposal generation, localization, and classification. Fifthly, the trained M-RCNN automatically detects the carious regions and encloses them in rectangles (Fig. 1E). Finally, the accuracy of cavity detection is analyzed (Fig. 1F). We also developed a graphical user interface (GUI) based dental carious region detection tool by employing the M-RCNN. The tool source, configuration, and installation information are publicly accessible from Zenodo Repository (https://doi.org/10.5281/zenodo.5680365). The researchers/practitioners can download the tool from the Zenodo repository and install it along with GUI on their personal computers by following the tool’s installation instructions.

#### Formalization

Let *D* be the dataset consisting of colored photographic *α*, X-ray radiographic *β*, and mixed (colored photographic and X-ray radio graphic) γ images dataset, given as *D* = {α, *β*, γ}, where $\alpha = \left\{ {\left\{ {cimg_1^x,cimg_1^y} \right\}, \ldots ,\left\{ {cimg_n^x,cimg_n^y} \right\}} \right\},\;$

$$\beta  = \left\{ {\left\{ {ximg_1^x,ximg_1^y} \right\}, \ldots ,\left\{ {ximg_n^x,ximg_n^y} \right\}} \right\},\>{\rm{and}}\>\gamma  = \left\{ {\left\{ {mimg_1^x,mimg_1^y} \right\}, \ldots ,\left\{ {mimg_n^x,mimg_n^y} \right\}} \right\}.\>$$

The $x > 0$ and $y > 0$ denote horizontal and vertical coordinates of an image, respectively.

Let ${D^l} \subset {\rm \; }D{\rm \; and\; }{D^t} \subset {\rm \; }D$ be the subset of *D*, used for model training and testing, respectively. Let ${D_s}$ be the dataset of enhanced images. To sharpen the carious regions in the images, we used 2^nd^ order derivative Laplacian filtration function on input image f(x, y). Similarly, we used function g(x, y) to obtain the sharpened image.

(1) $${\Delta ^2}f\left( {x,y} \right) = \displaystyle{{{\partial ^2}\; f\left( {x,y} \right)} \over {\partial {x^2}}} + \displaystyle{{{\partial ^2}\; f\left( {x,y} \right)} \over {\partial {y^2}}}.$$

(2) $$g\left( {x,y} \right) = \left\{ {\matrix{ {f\left( {x,y} \right) - {\Delta ^2}f\left( {x,y} \right)\; \; \; if\; {W_c} \lt 0} \cr {f\left( {x,y} \right) + {\Delta ^2}f\left( {x,y} \right)\; \; \; if\; {W_c} \gt 0} \cr } } \right..$$

where ${W_c}$ is the centralized pixel value. Using g(x, y), we obtain the enhanced images dataset given as ${D_s} = \mathop \sum \nolimits_{i = 1}^n g\left( {d_i^x,{\rm \; }d_i^y} \right)$, where $d \in D$. To train the model for the identification of carious regions, we fed the model with manually labeled images. For all images in ${D^l}$ (*i.e*., $\forall {D^l}){\rm \; }$ we used lblimg (https://github.com/tzutalin/labelImg) tool to draw rectangles R on the carious regions *e.g*., $R = \left\{ {{r_{1,{\rm \; } \ldots ,}}{r_n}} \right\},{\rm \; }$ where R specifies the rectangular coordinates given as $r = \left\{ {{x_{min,{\rm \; \; }}}{x_{max,{\rm \; }}}{\rm \; }{y_{min,{\rm \; }}}{\rm \; }{y_{max}}} \right\}.$ Each R is encoded in an *XML* file.

The significant aspect of the deep learning approach is automated features extraction. We used a *ResNet50* convolutional neural network consisting of 50 deep layers. The *ResNet50* takes an input image and transforms it into a vector of 2,048 dimensions. We obtained the image features dataset ${D^f}$ for all images in ${D^l}$, given as ${D^f} = \left\{ {\forall d \in {D^l}|{\rm \; }ResNet50{\rm \; }\left( d \right) \to \left( {{x_{1,{\rm \; } \ldots .,}}{x_{2048}}} \right)} \right\}{\rm \; where\; }x \in {\rm {\mathbb R}}$. Moreover, $\forall d \in {D^f}$, Region Proposal Network (RPN) creates an $n \times n$ sliding window ${S_{n \times n}}$ that convolves over *d*. The RPN takes *d* as an input and performs classification $cls$ and regression $reg$, given as $RPN\left( d \right) \to \left( {cls,reg} \right).$ The $cls$ generates 2k scores, predicting whether the ${S_{n \times n}}$ contains the carious region or not. The $reg$ generates 4*k* scores (x, y, w, h) that enclose the carious region in ${S_{n \times n}}$.

Let ${M_o}$ be the output of the model containing the localized image ${I_o}$, given as ${I_o} = \left\{ {{x_{min,\; \; }}\; {x_{max,\; }}\; {y_{min,\; }}\; {y_{max }}} \right\}$. The output of the model is considered valid if the target image ${I_t}$ and the annotated image ${I_r}$, where $r \in R$, have a defined threshold of Intersection over Union, given as:

(3) $$IoU = \displaystyle{{{I_o} \cap {I_r}} \over {{I_o} \cup {I_r}}}.$$

The region of interest (cavity) is only considered positive if the Intersection Over Union (IoU) of the predicted rectangle by *reg* and manually annotated rectangle *R* exceeds the ground-truth value of 0.6. Afterwards, we used precision and recall to evaluate the effectiveness of our model. For precision, 11-point interpolation ${{\rm P}_{11\_{\rm pt}}}\left( {\rm r} \right)$ is used. It is calculated at each recall level *r'*, by taking the maximum measured precision of *r*. After interpolating the precision-recall curve, the final precision is calculated by taking a sum of different rectangles. Let *k* be the number of rectangles, *N* be the total number of the images, and *i* be the image at $ith$ position. We compute precision measure as $P = \sum \left( {{r_{n + 1}} - r} \right).{{\rm P}_{11\_{\rm pt}}}{r_{r + 1}}$, where ${P_{{{11}_{{\rm pt}}}}}\left( {\rm r} \right) = {\rm ma}{{\rm x}_{{r}^{\prime} \ge r}}\ P\left( {{r}^{\prime}} \right)\;$and recall as $R = \displaystyle{1 \over N}\; \mathop \sum \nolimits_{i = 1}^k \left( {{r_i}} \right)$, where $P\left( {{r}^{\prime}} \right) \ge P\left( r \right)$.

#### System architecture

**Figure 2:**
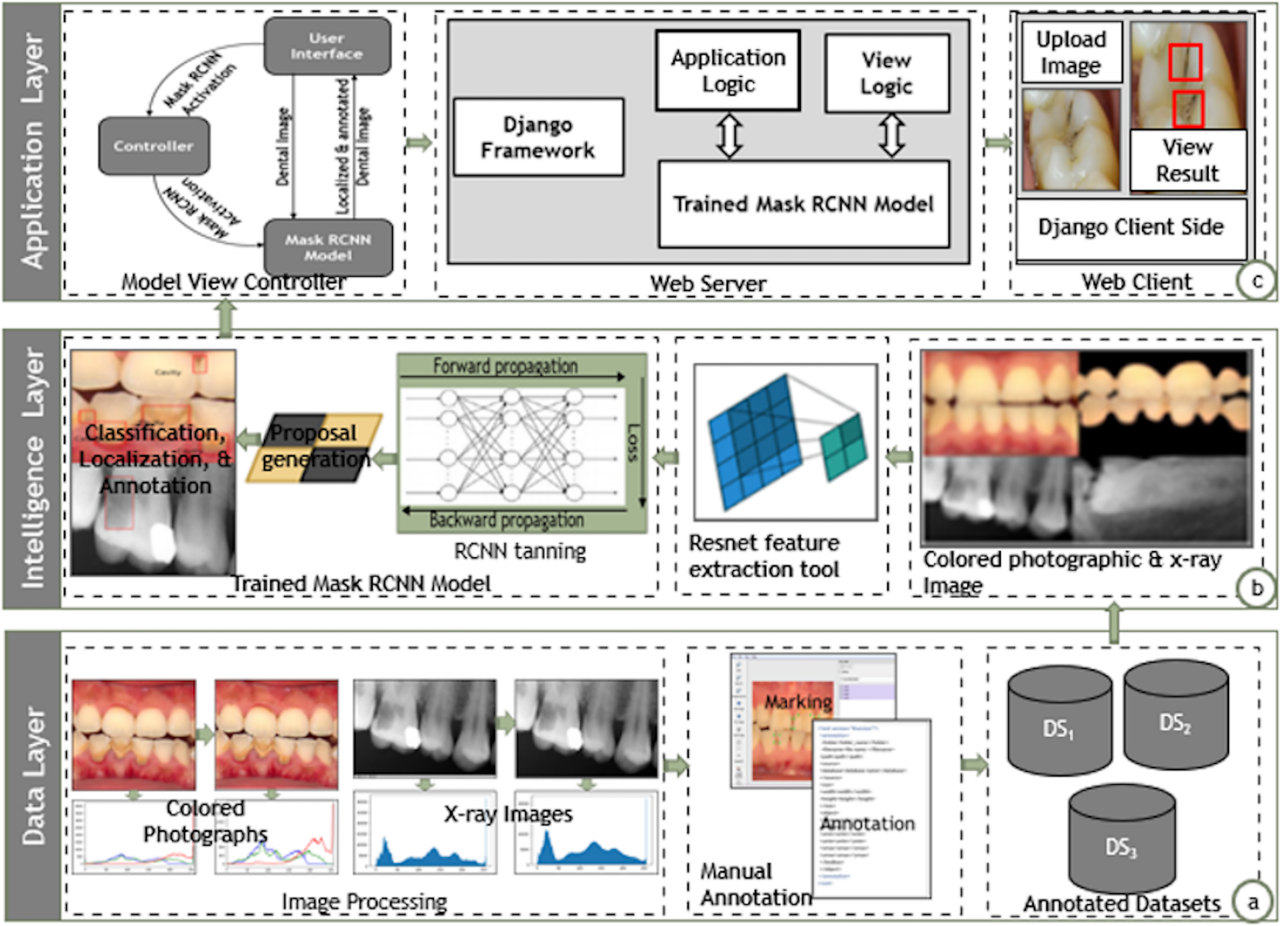
Architectural design of proposed Mask-RCNN approach.

A software architecture creates, interprets, analyzes, and designs solutions within a particular knowledge domain (May, 2011). The architecture decomposes domain problems into subsystems, layers, and integrated components. The architectural design envisions the systems in broader perspectives and elaborates the communication among the components distributed in non-overlapping layers, which may pertain to the functionalities associated with them (Richards, 2015; Perry & Wolf, 1992). We also suggested a layered-based architecture to enable the instantiation of the proposed approach. Fig. 2 depicts the architectural design of the proposed approach containing data, intelligence, and application layers.

##### Data layer

The data layer pertains to the details related to collection, normalization, and annotation of dental image datasets for further training and localization, as shown in Fig. 2A. The data layer annotates the mixed dental images collected from various sources. It performs necessary histogram equalization *via* sharpening filters to uniformly distribute the color and grey-scale intensities. The data layer further employed the *lblimg* tool to manually annotate the dental images with the support of human dental experts. The data layer finally stores the annotated images in three separate datasets containing the colored photographs, X-rays radiographs, and mixed dental images.

##### Intelligence layer

The intelligence layer provides training and prediction of dental carious regions as shown in Fig. 2. The intelligence layer takes annotated dental images from the three distinct datasets, *i.e*., colored, X-ray, and mixed. It further employs the ResNet feature extractor module to extract the high-level features from the dental images. The M-RCNN training module generates the RPN by employing the high-level features and annotations in the classification, localization, and annotation of dental carious regions. Ultimately, the intelligence layer enables the input of a dental image (colored photograph or X-ray radiograph) and localizes the carious regions.

##### Presentation layer

The application layer envisions the instantiation of the proposed approach over the real generic applicative scenarios, as shown in Fig. 2C, the presentation layer employs the Model View Controller (MVC) architecture to instantiate dental carious regions localization tool. The view is the front-end interface; the model is the trained M-RCNN over the dataset of mixed dental images (colored and X-ray images); the controller contains the logic to activate the M-RCNN model. The presentation layer provides a search engine that accepts an image and localizes the carious regions. The search engines enable the processing *via* the Django web framework. The application interface directly links the controller layer to trigger the M-RCNN model, which processes the dental image and localizes the carious region. The application interface displays the localized version of the dental image.

### Instantiation

The deep learning models are widely exploited in the detection of dental cavities. The basic CNN architecture considers VGG-16, Faster RCNN, YOLO, Mask RCNN, *etc*., as pre-trained deep learning models. The employment of the CNN models in dental healthcare and clinical setting is subject to their suitability and specifications (Ezhov et al., 2021; Koutsouri et al., 2013). The researchers commonly employed a convolutional neural network to diagnose diseases in dental images. However, they used VGG-16 as a pre-trained model in oral disease classifications (Prajapati, Nagaraj & Mitra, 2017). The YOLO framework employs a single CNN model and entire image to detect possible objects in images and regions of interest. The YOLO gives satisfactory results in bitewing dental images (Bayraktar & Ayan, 2021). The M-RCNN is an extension of Faster RCNN and is used to predict multiple regions of interest (Prados-Privado et al., 2020). Researchers, such as Zhu, Piao & Kim (2020) used M-RCNN to identify tooth regions in images instead of YOLO and Faster RCNN. Our investigation reveals the distinct implication of M-RCNN model in the detection of dental cavities *via* multiple image types.

This research proposed a generic approach that can localize carious regions in mixed (colored dental photographic and X-ray radiographic) images using the transfer learning approach with minimum supervision requirements from an expert (Jader et al., 2018). We employed a pre-trained M-RCNN model with the conjunction of preprocessing techniques (ResNet50 for feature extraction and classification purpose along with gradient descent). We exploited the laplacian sharpen filter to boost the carious regions’ pixels in dental images and renowned Scikit-image version 0.15.0 (python package built upon NumPy, SciPy, and matplotlib) for image processing since it contains several algorithms and works with several types of heterogeneous data structures. The preprocessing highlighted the dark regions; this enhanced the lighter pixels of dental images. We firstly considered transfer learning *via* feature extraction scheme since our dataset is heterogeneous, containing colored dental photographic and X-ray radiographic dental images. The M-RCNN is a pre-trained model for end-to-end learning on our custom dental dataset with ResNet50 for feature extraction. It is essential to note that the approach can also work on heterogeneous localization architectures. Afterward, region-based annotated images are passed to a pre-trained M-RCNN model to locate the carious region.

**Figure 3:**
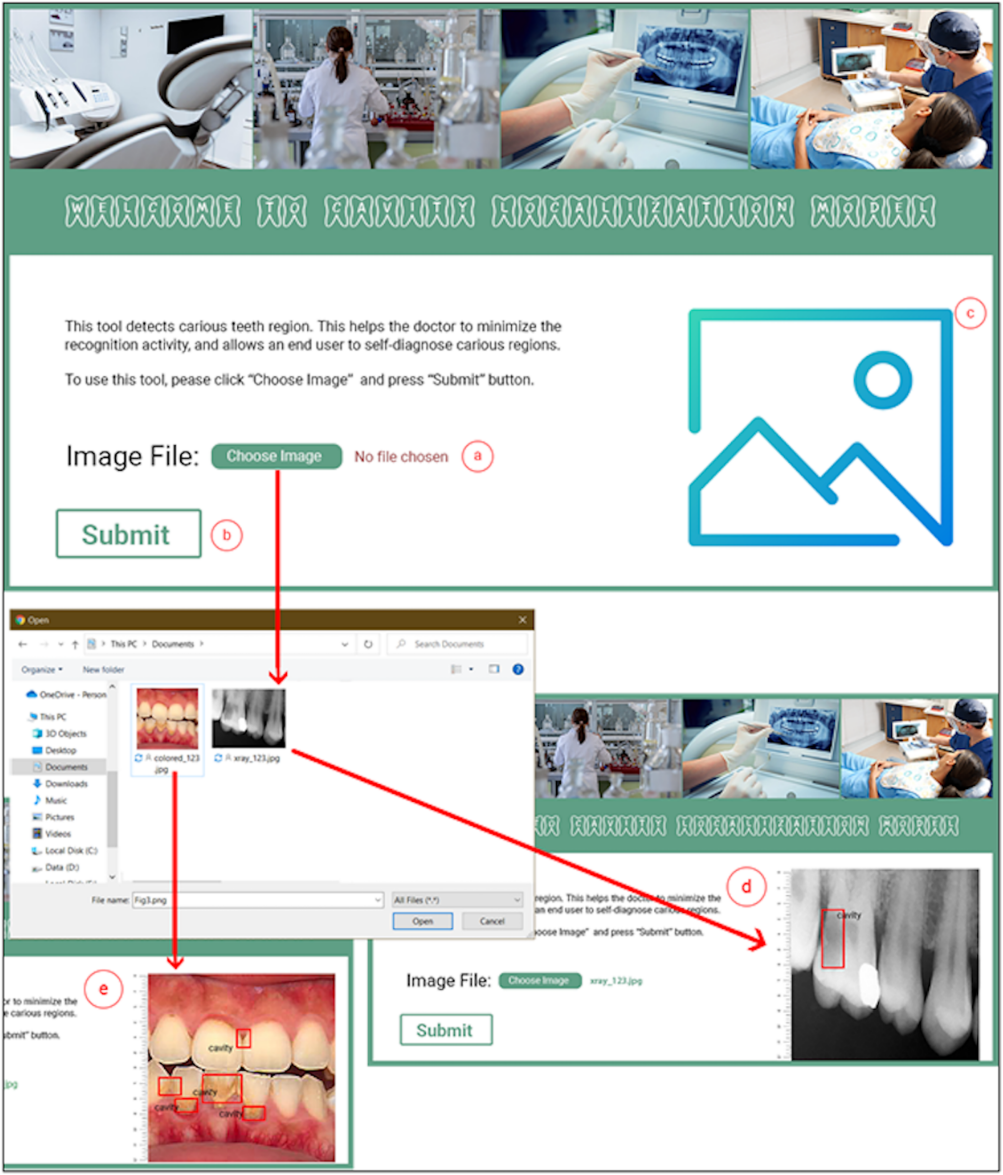
Proposed dental cavity detection tool: (A) image upload, (B) submit button, (C) output image area, (D) X-ray image output, and (E) colored image output.

The M-CRNN model was instantiated using the python programming language on an ordinary Intel Core (TM) i3- 4010U CPU @ 1.70 GHz, having 4.00 GB of RAM and Django web framework (https://www.djangoproject.com/). The Django web framework is considered a high-level python web development framework. We designed and developed a complete toolkit that facilitates a dentist in diagnosing carious teeth regions. Figure 3 depicts the interface details of the dental cavity detection and localization tool. The dental cavity detection tool helps the end-user to self-diagnose the carious tooth regions. Along with that, the tool facilitates in reducing the excessive workload from the dentists. The tool detects carious regions from colored photographic or X-ray radiographic images in three simple steps. In the first step, the user uploads a tooth image (Fig. 3A). Afterward, the user clicks the submit button (Fig. 3B). The uploaded picture is passed to the proposed model, and the detected localized carious region output image is displayed on the user interface for the input X-ray radiograph (Fig. 3D) or colored photographs (Fig. 3E).

## Experimental setup and results

### Dataset validation

We hired two qualified dentists ( ${D_1}$ and ${D_2}$) with more than 3 years of work experience to manually annotate carious regions using the lblimg tool. The dentists used bounding boxes to enclose each carious region. A perfect bounding box is the one that covers each carious region of a dental image. Afterwards, the model is trained disjointly on the annotated dataset generated by ${D_1}$ and ${D_2}$. We examined the correctness of the annotated dataset using two different values of Intersection over Union (IoU). This procedure was adopted to check the overlapping ratio of expected and truth rectangles. Average precision measure is used to validate the correctness of the annotated dataset. The dataset information and the results obtained are displayed in Table 2.

**Table 2:** Dataset validation scores between dentists (*D*_1_ and *D*_2_).

Dataset	Images		Dentist	Precision	
	Local	Public		IoU = 0.6	IoU = 0.7
X-ray	816	120	${D_1}$	84.45%	79.54%
			${D_2}$	84.41%	79.52%
Color	–	90	${D_1}$	95.75%	89.06%
			${D_2}$	95.06%	89.00%

Table 2 highlights the correctness and validity of our gathered and annotated datasets. The table depicts the comparison of 816 locally annotated instances of the X-ray radiographs and publicly available instances of 120 X-ray radiographs and 90 colored photographs. On average, a subtle difference of 4% between the scores ${D_1}$ and ${D_2}$ is observed. Hence, 96% of agreement exists between the dentists on the correctness of locally annotated and publicly available datasets of annotated images. It shows the high accuracy of the locally gathered and annotated dataset of dental images.

### M-RCNN model evaluation

We split our datasets into training and testing sets. The training dataset is used to train the proposed M-RCNN model. The testing dataset is used to evaluate the accuracy of the proposed trained M-RCNN model. We trained and tested the proposed M-RCNN model on the colored photographs, X-ray radiographs, and mixed images dataset seperately. The training and testing dataset split ratio was set respectively to 70:30 for colored photographic dataset and 80:20 for the X-ray radiographic and mixed datasets, the most commonly used dataset splitting standard in machine learning research (Wenzel, 2002). Due to the lack of a large number of the publically available colored dataset, we increased the training dataset size for the colored photographic dataset to produce realistic results. We assessed the performance of the proposed model using precision and recall measures over the colored photographic, X-ray radiographic, and mixed dental image datasets. The experimental parameters and results obtained are explained in subsequent sections.

#### M-RCNN model parameterization

We used ResNet50 for feature extraction and classification (Jader et al., 2018). The model learning rate is usually set to 0.1, 0.01, or 0.001. We set the learning rate of 0.01 to keep the minimum requirements of storage usage. The number of epochs was two, and the four batches of images were chosen. We achieved the best results using these parameters. Table 3 shows the parameter values used to train and test the proposed model. These model parameters are used for colored photographic, X-ray radiographic, and the mixed dataset of dental images.

**Table 3:** M-RCNN model configuration by employing colored, X-ray, and combined dental images dataset.

Configuration	Description
Learning rate	0.01
# of Epochs	2.0
Batch size	4.0

#### Empirical results

**Figure 4:**
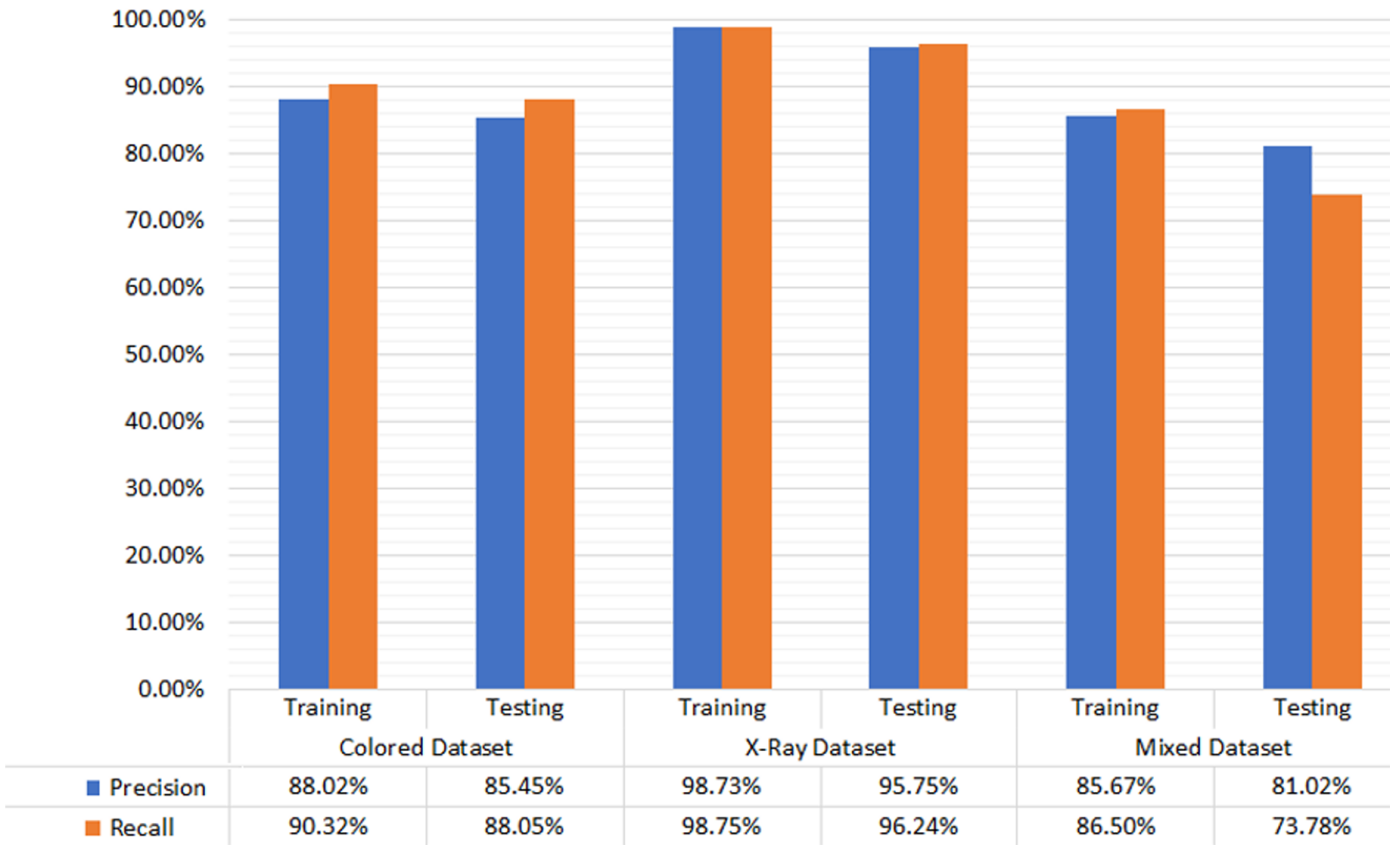
Evaluation results for color photographic, X-ray, and mixed datasets.

The empirical evaluation *via* precision and recall gives satisfactory results. The difference in the precision and recall measures on different types of dental image datasets is marginal. Hence the proposed M-RCNN is equally applicable to the different types of dental images datasets. Figure 4 shows the testing and training dataset results. In the colored photographic dataset, our approach achieved 88.02% and 85.45% precision in training and testing, respectively. Our approach gained 90.32% and 88.05% recall in the training and testing dataset, respectively. In the X-ray radiographic dataset, the precision in training and testing is 98.73% and 95.75%, respectively. The recall in training and testing is 98.75% and 96.24%, respectively. We achieved 85.67% and 81.02% precision in training and testing in the mixed dataset, respectively. We reached 86.5% and 73.78% recall in training and testing, respectively.

The dental carious regions’ localization results of the M-RCNN model are relatively similar. The difference over distinct datasets containing colored photographic, X-ray radiographic, and mixed dental images is marginal. However, results are somewhat compromised over the separate datasets. The results can be fine-tuned by taking the optimized dental images and by applying different hyper-parameters. We examined the correctness of colored photographic, X-ray radiographic, and mixed images, *i.e*., $D{S_1}$, $D{S_2}$, and $D{S_3}$, respectively, by employing different values of IoU. We adopted the procedure to check the overlapping ratio of correct results over other datasets by exploiting the proposed M-RCNN model. The precision and recall measures are used to validate the correctness of the distinct (colored photographic, X-ray radiographic, and mixed) annotated dataset. Table 4 depicts the dataset information and the corresponding results.

**Table 4:** Correctness validation scores in datasets DS1, DS2, and DS2 containing colored photographic, X-ray radiographic, and mixed dental images, respectively by employing M-RCNN model in terms of precision (P) and recall (R) measures.

	DS1: Colored dental dataset		DS2: X-ray dental dataset		DS3: Mixed dataset	
	P	R	P	R	P	R
@IoU = 0.6	95.75	96.24	84.45	85.05	81.02	83.78
@IoU = 0.7	89.06	92.09	79.54	80.45	76.02	78.78

### The tool’s evaluation

We measured the usability of our designed and developed dental cavity detection tool using a standard System Usability Scale (SUS) instrument given in literature (Bangor, Kortum & Miller, 2009; Brooke, 2021). The SUS instrument consists of 10-items to uncover the diverse usability dimensions in the design of interactive tools. The instrument is available at a web source (https://www.usability.gov/how-to-and-tools/methods/system-usability-scale.html). According to the literature, a minimum of 12–14 participants are required to reveal satisfactory results (Tullis & Stetson, 2004; Nielsen & Landauer, 1993). Therefore, we invited 17 participants. Among the 17 participants, 3 were dentists, 4 were dentist assistants, and 10 were final-year dental students. The evaluation was conducted in a silent room, having a workstation equipped with necessary hardware and software support.

The participants were asked to perform a complex scenario containing the structured sub-tasks after giving them a brief introduction and demonstration of the dental cavity detection tool. In the scenario, the users were asked to (i) capture a dental photograph image having a cavity *via* ordinary camera, (ii) upload a dental image over the dental cavity detection tool, (iii) analyze the carious region localized by the dental cavity detection tool, (iv) capture a dental photograph image having no cavity *via* ordinary camera, (v) upload a dental image over the dental cavity detection tool, and (vi) analyze the suggested results and exit the dental cavity detection tool.

Upon completing the mentioned scenario, the participants were asked to give responses *via* the SUS instrument. Table 5 highlights the SUS experimental results. According to the SUS analysis, the dentists were the most satisfied with the tool, with the SUS scale score obtained 90.8%. Then, the students were the most satisfied, with a SUS scale score equal to 85.3%. Finally, the dentist assistants also rated the proposed dental cavity detection tool, and the SUS score obtained 84.4%. The usability ratings provided by each participant are shown in Table 5. Overall, the proposed dental cavity detection tool achieved an 86.82% SUS scale score, graded as A+ grade, and falls in the top 95–100 percentile software category in terms of usability (Lewis & Sauro, 2018).

**Table 5:** SUS analysis of dental cavity detection tool.

Questions	(D)entist			(A)ssistant				(S)tudent									
	D1	D2	D3	A1	A2	A3	A4	S1	S2	S3	S4	S5	S6	S7	S8	S9	S10
I think that I would like to use this tool	4	5	5	5	5	4	4	4	4	4	5	4	5	4	4	4	4
I found the tool unnecessarily complex	1	2	2	1	2	2	1	1	2	1	2	2	1	2	2	2	1
I thought the tool was easy to use	4	5	5	5	5	4	5	5	4	4	5	4	4	5	5	5	5
Support of a technical person is required to use this tool	1	1	1	2	1	2	2	2	3	2	2	2	2	1	1	2	2
This tool is well integrated	4	5	5	3	5	4	4	4	4	4	4	4	5	5	5	5	4
I found inconsistency in this tool	2	1	1	1	1	2	1	1	2	1	1	2	1	1	1	1	1
People would learn to use this tool quickly	4	5	5	5	5	4	4	4	4	4	5	4	5	4	4	4	4
I found the tool very awkward to use	1	1	3	1	1	2	2	1	1	1	1	2	1	2	2	1	1
I felt very confident using the tool	4	5	4	5	5	4	4	5	5	5	5	4	5	5	5	4	4
I needed to learn a lot of things before using it	1	1	1	3	3	2	2	2	3	2	2	2	2	2	2	2	1
Score	85	97.5	90	87.5	92.5	75	82.5	87.5	75	85	90	75	92.5	87.5	87.5	85	87.5
**Average**	90.83			84.38				85.25									

## Comparison and implications

Our proposed M-RCNN model used a transfer learning approach to run on a minimum hardware configuration. The proposed approach is compared with state-of-the-art approaches, *i.e*., K-means, J48, Random Forest, SVM, Deep Conv Net, pre-trained model VGG16, Inception CNN, CNN, and U-Net in terms of precision. The comparison of the proposed M-RCNN with the baseline approaches is summarized in Table 6. The M-RCNN approach outperforms the previous approaches on the colored photographic dataset. However, the proposed approach still has room for improvement in detecting carious regions from X-ray radiographs.

**Table 6:** Comparison of proposed M-RCNN model approach with state-of-the-art approaches discussed in recent years.

Dataset	Authors	Technique	Dental image instances		Results @ Precision/Accuracy (%)
Colored photographs	Gonsalves (2017)	K-means		60	P: 80.00
	Moutselos et al. (2019)	J48		91	P: 83.00
	Prados-Privado et al. (2020)	Random Forest		88	A: 86.30
	Navarro et al. (2019)	SVM		45	P: 84.00
	Berdouses et al. (2015)	Deep Conv Net		3,932	A: 80.00
	Proposed	M-RCNN		90	P: 88.02
X-ray/grayscale radiographs	Kumar, Bhadauria & Singh (2021)	SVM		100	P: 86.70
	Lee et al. (2018)	VGG16 Model		125	P: 88.46
	Koutsouri et al. (2013)	Inception CNN		3,000	A: 82.00
	Ghaedi et al. (2014)	CNN		217	A: 84.60
	Vashishth, Kaushal & Srivastava (2014)	U-Net		3,686	A: 80.00
	Proposed	M-RCNN		936	A: 95.75
Mixed	Proposed	M-RCNN		210	P: 81.02

Moreover, several dental images exist, *e.g*., panoramic, bitewings, periapical, X-ray, and camera taken images. While our approach utilizes X-ray and camera-taken dental images, the proposed approach is yet to be tested on the other specific types of images. It is due to the lack of a publically available dataset of panoramic, bitewings, and periapical dental images. We envision the proposed approach as an optimistic baseline for further implementation of divergent and heterogeneous large datasets of dental images with our promising initial results.

The previous approaches classified the carious regions globally, resulting in increased accuracy scores. The utilization of more advanced R-CNN models can increase dental caries detection and localization accuracy at the cost of increased computational requirements. The model developed in this research requires specific software and hardware configuration. However, our goal is to provide a complete package with minimum software and hardware configuration. Therefore, we developed a dental cavity detection and localization tool that can run on any ordinary computer hardware and be used by experts and novice users alike.

## Conclusion and future work

Deep learning is an emerging area of medical research that has shown significant results in detecting and diagnosing carious regions. People with dental problems require proper consultancy from dentists. The manual diagnostic approaches may sometimes lead to misdiagnosis. The advancement in artificial intelligence helps dentists/researchers to develop useful tools for detecting carious regions from dental images. Contributions of our work mainly focused on three significant aspects. Firstly, we designed the dental images dataset, which the medical experts evaluate to ensure correctness and validity. The dataset can serve as a baseline for future dental researches. Secondly, we proposed a transfer learning approach to detect the dental carious region from colored photographic, X-ray radiographic, and mixed datasets (colored photographs and X-ray radiographs). We were the first to investigate this type of deep learning neural model optimized to detect and localize carious regions in real-time on a personal computer. Thirdly, we designed and developed a visually appealing and easy-to-use tool that allows dentists and naïve users to detect and localize dental carious regions. The medical experts and general audience evaluated the proposed tool, passed the system usability test with an A+ grade, and achieved highly usable software’s top 95 percentile category.

Moreover, the proposed deep neural model was empirically evaluated individually on the colored photographic and X-ray radiographic images. We compared the precision and recall of our proposed approach with state-of-the-art approaches. The empirical testing was also performed on the mixed dataset of colored photographic and X-ray radiographic images. The precision was 95.75%, 84.45%, and 81.02% over the colored photographic, X-ray radiographic, and mixed datasets. Similarly, the recall was 96.24%, 85.05%, and 83.78% over the colored photographic, X-ray radiographic, and mixed datasets, respectively, surpassing various past approaches. It is essential to mention that the previous approach reveals similar results over the distinct image type (*i.e*., colored photographic and X-ray radiographic, panoramic images, *etc*.). However, a hybrid/mixed approach trained over the mixed dataset containing the colored photographs and X-ray photographs is not discussed previously in the literature.

In the future, we will extend the functionality of the dental caries detection tool. We look forward into collecting and growing the proposed methodology on panoramic, bitewings, and periapical dental images. We have intentions to design and develop a comprehensive system that will be a hybrid of connectionist and symbolic learning models capable of detecting early lesions and further recommend a suitable course of action for dental caries. In addition to this, we will also deploy the approach over smartphones to facilitate real-time detection, localization, and recommendation to treat dental carious regions.

## Supplemental Information

10.7717/peerj-cs.888/supp-1 Supplemental Information 1 SUS Scale Scores.

10.7717/peerj-cs.888/supp-2 Supplemental Information 2 SUS RAW SCORE.

10.7717/peerj-cs.888/supp-3 Supplemental Information 3 USER demographics.

10.7717/peerj-cs.888/supp-4 Supplemental Information 4 Questionnaire.

10.7717/peerj-cs.888/supp-5 Supplemental Information 5 Evaluation Steps.

## Additional Information and Declarations

### Data Availability

The following information was supplied regarding data availability: The raw data is available at Zenodo: Umer Rashid. (2021). A hybrid Mask RCNN based tool to localize dental cavities from real-time mixed photographic images (Version 1). Zenodo. https://doi.org/10.5281/zenodo.5347953.
