# Supplementary material for: A hybrid mask RCNN-based tool to localize dental cavities from real-time mixed photographic images
Source: PeerJ Comput Sci. 2022 Feb 18;8:e888. doi: 10.7717/peerj-cs.888 (PMC9044255; doi:10.7717/peerj-cs.888)
Supplement: Supplemental Information 1 [file peerj-cs-08-888-s001.pdf]

Scale Scores										
	Q1	Q2	Q3	Q4	Q5	Q6	Q7	Q8	Q9	Q10	Score Value
D1	4	1	4	1	4	2	4	1	4	1	85
D2	5	2	5	1	5	1	5	1	5	1	97.5
D3	5	2	5	1	5	1	4	3	5	1	90
A1	5	1	5	2	3	1	5	1	5	3	87.5
A2	5	2	5	1	5	1	5	1	5	3	92.5
A3	4	2	4	2	4	2	4	2	4	2	75
A4	4	1	5	2	4	1	4	2	4	2	82.5
S1	4	1	5	2	4	1	5	1	4	2	87.5
S2	4	2	4	3	4	2	5	1	4	3	75
S3	4	1	4	2	4	1	5	1	4	2	85
S4	5	2	5	2	4	1	5	1	5	2	90
S5	4	2	4	2	4	2	4	2	4	2	75
S6	5	1	4	2	5	1	5	1	5	2	92.5
S7	4	2	5	1	5	1	5	2	4	2	87.5
S8	4	2	5	1	5	1	5	2	4	2	87.5
S9	4	2	5	2	5	1	4	1	4	2	85
S10	4	1	5	2	4	1	4	1	4	1	87.5
Average Score											86.03
