# Supplementary material for: A hybrid mask RCNN-based tool to localize dental cavities from real-time mixed photographic images
Source: PeerJ Comput Sci. 2022 Feb 18;8:e888. doi: 10.7717/peerj-cs.888 (PMC9044255; doi:10.7717/peerj-cs.888)
Supplement: Supplemental Information 2 [file peerj-cs-08-888-s002.pdf]

Questionaire Feedback											
	Q1	Q2	Q3	Q4	Q5	Q6	Q7	Q8	Q9	Q10	Raw Score
D1	4	1	4	1	2	4	1	4	1	4	45
D2	5	2	5	1	1	5	1	5	1	5	37.5
D3	5	2	5	1	1	5	3	4	1	5	45
A1	5	1	5	2	1	3	1	5	3	5	47.5
A2	5	2	5	1	1	5	1	5	3	5	42.5
A3	4	2	4	2	2	4	2	4	2	4	45
A4	4	1	5	2	1	4	2	4	2	4	47.5
S1	4	1	5	2	1	4	1	5	2	4	42.5
S2	4	2	4	3	2	4	1	5	3	4	40
S3	4	1	4	2	1	4	1	5	2	4	40
S4	5	2	5	2	1	4	1	5	2	5	40
S5	4	2	4	2	2	4	2	4	2	4	45
S6	5	1	4	2	1	5	1	5	2	5	37.5
S7	4	2	5	1	1	5	2	5	2	4	42.5
S8	4	2	5	1	1	5	2	5	2	4	42.5
S9	4	2	5	2	1	5	1	4	2	4	40
S10	4	1	5	2	1	4	1	4	1	4	42.5
