# Supplementary material for: A hybrid mask RCNN-based tool to localize dental cavities from real-time mixed photographic images
Source: PeerJ Comput Sci. 2022 Feb 18;8:e888. doi: 10.7717/peerj-cs.888 (PMC9044255; doi:10.7717/peerj-cs.888)
Supplement: Supplemental Information 3 [file peerj-cs-08-888-s003.pdf]

User Demographic Details								
Uid	Name	Age	Gender	Manual Assessment Expirience(Years)	Manual tools to assess Dental Images	Automatic Assessment Expirience	Well Known dental Images	Automatic Tool for dental Image Processing
D1	Ghazala	42	Female	15	Mirror, dental Explorer	10 Years	X-Ray, Digital Colored	Logicon
D2	Imran Rasheed	38	Male	11	Mirror, Dental Explorer	0	X-Ray	Nill
D3	Ali Imram	43	Male	15	Mirror, Dental Explorer	10 Years	X-Ray, Colored	Logicon
A1	Naier Jamal	20	Male	1	Examination Set	0	X-Ray	Nill
A2	Sundas Javed	22	Female	3	Examination Set	0	X-Ray	Nill
A3	Anosha Sajjad	23	Female	2	Examination Set	0	X-Ray	Nill
A4	Tariq Hussain	18	Male	1	Examination Set	0	X-Ray	Nill
S1	Sidra Nasir Qazi	25	Female	0	Mirror, Dental Explorer	0	X-Rays, Colored	Nill
S2	Salka Urooj	25	Female	0	Mirror, Dental Explorer	0	X-Rays, Colored	Nill
S3	Bakhtawar Aziz	24	Female	0	Mirror, Dental Explorer	0	X-Rays, Colored	Nill
S4	Kiren Shambleed	26	Female	0	Mirror, Dental Explorer	0	X-Rays, Colored	Nill
S5	Anosha Sajid	25	Female	0	Mirror, Dental Explorer	0	X-Rays, Colored	Nill
S6	Wajih Kanwal	25	Female	0	Mirror, Dental Explorer	0	X-Rays, Colored	Nill
S7	Huria Javed	25	Female	0	Mirror, Dental Explorer	0	X-Rays, Colored	Nill

S8	Lalarukh Abbasi	24	Female	0	Mirror, Dental Explorer	0	X-Rays, Colored	Nill
S9	Farheen Sajid	25	Female	0	Mirror, Dental Explorer	0	X-Rays, Colored	Nill
S10	Hadia Azeem	25	Female	0	Mirror, Dental Explorer	0	X-Rays, Colored	Nill
