# Supplementary material for: A hybrid mask RCNN-based tool to localize dental cavities from real-time mixed photographic images
Source: PeerJ Comput Sci. 2022 Feb 18;8:e888. doi: 10.7717/peerj-cs.888 (PMC9044255; doi:10.7717/peerj-cs.888)
Supplement: Supplemental Information 4 [file peerj-cs-08-888-s004.pdf]

SUS Questionnaire (adapted as an instrument [1][2]) to check the usability of the proposed dental cavity detection tool

Q. No	Statement	Strongly disagree 1	2	3	4	Strongly agree 5
1	I think that I would use this tool frequently.					
2	I found the tool unnecessarily complex.					
3	I thought the tool is easy to use.					
4	I think, I need a technical person to use this tool.					
5	I thought, there exists a lot of inconsistency in tool.					
6	I found that many functions in tool were well integrated.					
7	I found awkward to use the tool.					
8	I felt very confident to use the tool.					
9	I needed to learn more before using the tool.					
10	The tool is effective to use in medical field.					

1. Bangor, P. Kortum and J. Miller, "Determining what individual sus scores mean: Adding an adjective rating scale", *Journal of usability studies, vol.* 4, no. 3, pp. 114-123, 2009.
2. J. Brooke, "Sus: Usability evaluation in industry",189, 1996.
