# Supplementary material for: A hybrid mask RCNN-based tool to localize dental cavities from real-time mixed photographic images
Source: PeerJ Comput Sci. 2022 Feb 18;8:e888. doi: 10.7717/peerj-cs.888 (PMC9044255; doi:10.7717/peerj-cs.888)
Supplement: Supplemental Information 5 [file peerj-cs-08-888-s005.pdf]

Evaluation Tasks

Task Steps: Participants are asked to perform via out tool.

1. Select dental image having cavity.
2. Upload dental image.
3. Analyze the carious region localized by model.
4. Select sound dental image (having no cavity)
5. Uplaod dental image
6. Analyze the popup and close it.
